# The Role of the Physician Assistant in Oncology

**DOI:** 10.6004/jadpro.2012.3.1.1

**Published:** 2012-01-01

**Authors:** Sarah Daniel

**Affiliations:** Ms. Daniel is a physician assistant at the University of Arizona Cancer Center in Tucson.

The dreaded phone call. It is a Friday afternoon, and I am sitting at my desk at the University of Arizona Cancer Center trying to decide the best way to deliver some very bad news to a lovely patient with resected pancreatic cancer. Mrs. X is a 60-year-old woman who took excellent physical care of herself all her life. She had no health problems until she was diagnosed with locally advanced pancreatic cancer. She completed neoadjuvant combined-modality therapy followed by a Whipple resection and postoperative adjuvant chemotherapy. I saw her in clinic for a follow-up visit yesterday, but her CA 19-9 level hadn’t come back yet. She was doing wonderfully from a clinical standpoint and expressed excitement about travel plans. When I received the results this morning, her level was 918 U/mL—a marked increase from her previously normal value. I was disappointed to see this and had been putting off making the follow-up phone call all day. I had promised Mrs. X that I would call her with the results. I knew full well that she would understand the implications of this situation.

I have been practicing in oncology for almost 10 years. Unlike many other clinicians who practice in oncology, I did not have an initial inspiration or personal reasons to work in this field. In 2001, two weeks after I had graduated from PA school, the entire first floor of our Houston home was destroyed by tropical storm Allison. As I watched our furniture floating in four feet of water, my husband broke even worse news: we did not have flood insurance. I desperately needed a job. My husband was in surgical residency at the time and suggested I apply to an open position at MD Anderson Cancer Center. I was blessed with a job offer and knew shortly after I started that I was going to love working in this field. I practiced for five years in the GI medical oncology department at MD Anderson, and then spent two years at Boston Medical Center working in hematology as a stem cell transplant coordinator. When my husband became an attending physician, we relocated to Tucson.

I currently practice at the University of Arizona Cancer Center in Tucson. The University of Arizona Cancer Center was founded in 1976 and has a history of excellence in research and community outreach. The University of Arizona Cancer Center is the only NCI-designated Cancer Center in the state of Arizona. The main cancer center building is a beautiful, free-standing 82,000 square foot building that has lovely meditation gardens and an excellent design for patient care. I am one of five advanced practitioners in our building, which provides outpatient care services. Our team meets monthly to discuss practice-related issues. I participate on a well-designed rapid response team that responds to any emergent situation that arises in our building. I currently have one designated supervising physician. I view my role as a PA as one that enhances the physician-patient relationship and provides higher satisfaction to the patients in our practice.

The patients I see routinely are those with esophageal, gastric, hepatocellular, pancreatic, neuroendocrine, and colorectal cancers, as well as cancers of unknown primary origin. I also see sick walk-in patients as needed with a wide variety of diagnoses. I practice in a team-centered approach, simultaneously in clinic with an attending physician, and also have independent clinics. I perform routine history and physical exams, order and interpret diagnostic tests, address concerns in the infusion unit, and write subsequent chemotherapy orders. I spend a large amount of clinical practice time managing issues that affect our patients such as depression, anxiety, insomnia, pain management, neuropathy, fatigue, mucositis, nausea, vomiting, diarrhea, constipation, and neutropenia. I believe PAs can also make a positive impact on a center’s research program, as research is the key to improving patient care. As the University of Arizona Cancer Center has numerous clinical trials available, I routinely identify patients for protocols and assist in enrollment.

Drawing on my 10+ years in practice, I believe there are certain essential elements that contribute to the success of a provider in oncology. Continually staying up to date on the recent oncology literature and clinical trials, maintaining solid internal medicine skills, and being able to deliver bad news in a genuinely empathetic manner are essential. I spend a large amount of time providing patient education regarding the natural history of GI tract cancers and potential treatment options and toxicities. I feel that patients will have a decreased level of anxiety if they have a plan to manage potential problems and they understand the goals of their treatment.

Prior to having two children, I was involved in numerous professional activities and committees. I have slowly tried to increase my involvement in more nonclinical endeavors. I recently have become more active in the Association of Physician Assistants in Oncology (APAO). I feel this is an organization with a clear mission: to promote the utilization of physician assistants in the delivery of the best possible care available to people with cancer and related diseases. I am currently the co-chair for the next APAO annual national conference in Scottsdale, Arizona, in the fall of 2012.

Back to Friday afternoon. I finally picked up the phone and made the call I was dreading. Mrs. X took the news with her usual grace, but I could sense her understandable fear and sadness. We agreed that we would obtain CT scans and a follow-up visit with our team as soon as possible to obtain a solid understanding of the situation.

In oncology we have our successes, but we have many failures as well. I wish I could say that I don’t think about my patients and their families when I lie in bed at night. I have realized that even if we do not always have the cure we so desperately desire, we do help our patients through what are most likely the toughest challenges of their lives. For that reason, practicing in oncology will remain one of my greatest passions.

